# (*E*)-2-Bromo­benzaldehyde oxime

**DOI:** 10.1107/S1600536811032211

**Published:** 2011-08-17

**Authors:** Afsaneh Zonouzi, Roghieh Mirzazadeh, Seik Weng Ng

**Affiliations:** a Department of Chemistry, College of Science, University of Tehran, PO Box 14155-6455 Tehran, Iran; b Department of Chemistry, University of Malaya, 50603 Kuala Lumpur, Malaysia; c Chemistry Department, Faculty of Science, King Abdulaziz University, PO Box 80203 Jeddah, Saudi Arabia

## Abstract

The configuration of the C=N double bond of the title compound, C_7_H_6_BrNO, is *E*; the non-H atoms are approximately coplanar (r.m.s. deviation = 0.038 Å). In the crystal, pairs of mol­ecules are linked by a pair of O—H⋯N hydrogen bonds about a center of inversion, generating hydrogen-bonded dimers.

## Related literature

For the synthesis, see: Jin *et al.* (2010 ▶). For the spectroscopic differentiation between *E* and *Z* isomers, see: Schnekenburger (1973 ▶). For reactions that produce 5-isoxazolpenicillins, see: Wang *et al.* (2007 ▶).
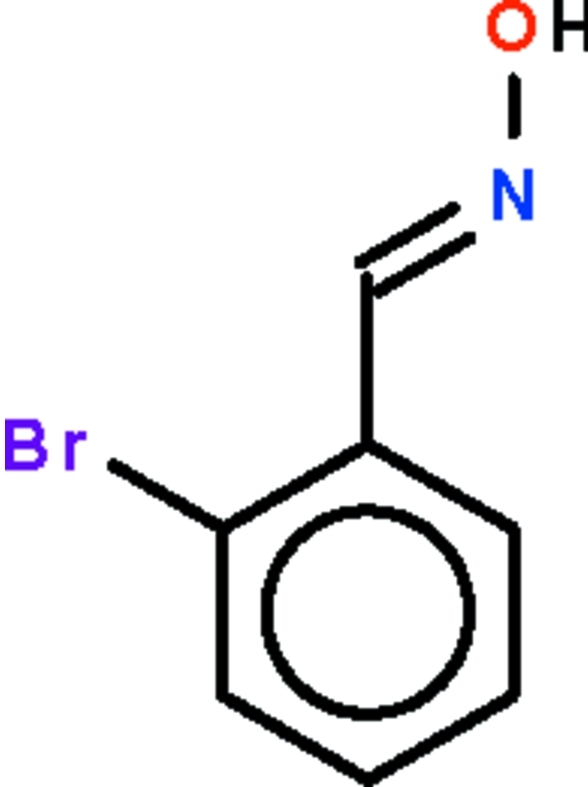


## Experimental

### 

#### Crystal data

C_7_H_6_BrNO
*M* *_r_* = 200.04
Monoclinic, 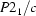

*a* = 7.7403 (2) Å
*b* = 4.0012 (1) Å
*c* = 23.2672 (5) Å
β = 98.810 (2)°
*V* = 712.09 (3) Å^3^
*Z* = 4
Cu *K*α radiation
μ = 7.25 mm^−1^
*T* = 100 K
0.20 × 0.15 × 0.10 mm

#### Data collection

Agilent SuperNova Dual diffractometer with an Atlas detector
Absorption correction: multi-scan (*CrysAlis PRO*; Agilent, 2010 ▶) *T*_min_ = 0.325, *T*_max_ = 0.531
4949 measured reflections
1421 independent reflections
1411 reflections with *I* > 2σ(*I*)
*R*_int_ = 0.017

#### Refinement

*R*[*F* ^2^ > 2σ(*F* ^2^)] = 0.022
*wR*(*F* ^2^) = 0.059
*S* = 1.06
1421 reflections
95 parameters
H atoms treated by a mixture of independent and constrained refinement
Δρ_max_ = 0.39 e Å^−3^
Δρ_min_ = −0.49 e Å^−3^

### 

Data collection: *CrysAlis PRO* (Agilent, 2010 ▶); cell refinement: *CrysAlis PRO*; data reduction: *CrysAlis PRO*; program(s) used to solve structure: *SHELXS97* (Sheldrick, 2008 ▶); program(s) used to refine structure: *SHELXL97* (Sheldrick, 2008 ▶); molecular graphics: *X-SEED* (Barbour, 2001 ▶); software used to prepare material for publication: *publCIF* (Westrip, 2010 ▶).

## Supplementary Material

Crystal structure: contains datablock(s) global, I. DOI: 10.1107/S1600536811032211/bt5606sup1.cif

Structure factors: contains datablock(s) I. DOI: 10.1107/S1600536811032211/bt5606Isup2.hkl

Supplementary material file. DOI: 10.1107/S1600536811032211/bt5606Isup3.cml

Additional supplementary materials:  crystallographic information; 3D view; checkCIF report

## Figures and Tables

**Table 1:** Hydrogen-bond geometry (Å, °)

*D*—H⋯*A*	*D*—H	H⋯*A*	*D*⋯*A*	*D*—H⋯*A*
O1—H1⋯N1^i^	0.86 (3)	1.98 (3)	2.802 (2)	159 (3)

Symmetry code: (i) 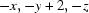.

**Fig. 1.**
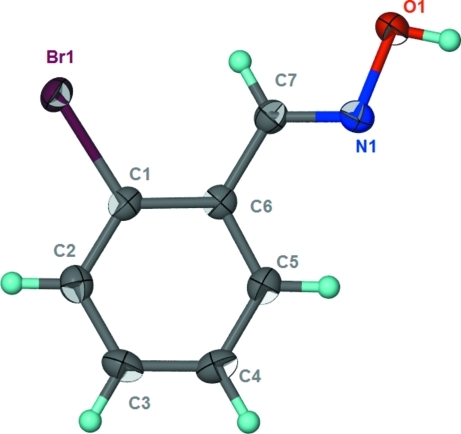
Anisotropic displacement ellipsoid plot (Barbour, 2001) of C7H6BrNO at the 70% probability level; hydrogen atoms are drawn as spheres of arbitrary radius.

## supplementary crystallographic information

### Comment

(2-Bromophenyl)methanoxime can be converted to 5-isoxazolpenicillins (Wang *et al.*, 2007); the compound exists into a *E* and a *Z* configuration with respect to the carbon-nitrogen double-bond; mixtures can be differentiated by their UV spectra (Schnekenburger, 1973). A recent study reported the synthesis of the *E* isomer (Scheme I) without the use of a metal-salt catalyst (Jin *et al.*, 2010). Zinc chloride is used in this study to give the compound in high yield. The non-H atoms are co-planar (Fig. 1); two molecules are linked by an O–H···N bond about a center-of-inversion to generate a hydrogen-bonded dimer (Table 1).

### Experimental

2-Bromobenzaldehyde (1.0 mmol, 184 mg), 50% hydroxylamine (3.0 mmol, 0.18 ml) and hydrated zinc chloride (0.2 mmol) were heated at 373 K for half an hour. The progress of reaction was monitored by TLC (ethyl acetate / *n*- hexane 1/3). The product was purified by column chromatography on silica gel, with ethanyl acetate/*n*-hexane (1/4) as co-solvent. Colorless were obtained by using ethyl acetate as solvent for recrystallization, m.p. 363 K (yield 90%).

### Refinement

Carbon-bound H-atoms were placed in calculated positions [C—H 0.95 Å, *U*_iso_(H) 1.2*U*_eq_(C)] and were included in the refinement in the riding model approximation.

The hydroxy H-atom was located in a difference Fouier map and was refined.

### Crystal data

**Table:**

C_7_H_6_BrNO	*F*(000) = 392
*M**_r_* = 200.04	*D*_x_ = 1.866 Mg m^−^^3^
Monoclinic, *P*2_1_/*c*	Cu *K*α radiation, λ = 1.54184 Å
Hall symbol: -P 2ybc	Cell parameters from 3601 reflections
*a* = 7.7403 (2) Å	θ = 3.8–74.0°
*b* = 4.0012 (1) Å	µ = 7.25 mm^−^^1^
*c* = 23.2672 (5) Å	*T* = 100 K
β = 98.810 (2)°	Block, colorless
*V* = 712.09 (3) Å^3^	0.20 × 0.15 × 0.10 mm
*Z* = 4	

### Data collection

**Table:**

Agilent SuperNova Dual diffractometer with an Atlas detector	1421 independent reflections
Radiation source: SuperNova (Cu) X-ray Source	1411 reflections with *I* > 2σ(*I*)
Mirror	*R*_int_ = 0.017
Detector resolution: 10.4041 pixels mm^-1^	θ_max_ = 74.2°, θ_min_ = 3.9°
ω scans	*h* = −8→9
Absorption correction: multi-scan (*CrysAlis PRO*; Agilent, 2010)	*k* = −4→4
*T*_min_ = 0.325, *T*_max_ = 0.531	*l* = −28→26
4949 measured reflections	

### Refinement

**Table:**

Refinement on *F*^2^	Primary atom site location: structure-invariant direct methods
Least-squares matrix: full	Secondary atom site location: difference Fourier map
*R*[*F*^2^ > 2σ(*F*^2^)] = 0.022	Hydrogen site location: inferred from neighbouring sites
*wR*(*F*^2^) = 0.059	H atoms treated by a mixture of independent and constrained refinement
*S* = 1.06	*w* = 1/[σ^2^(*F*_o_^2^) + (0.0373*P*)^2^ + 0.6889*P*] where *P* = (*F*_o_^2^ + 2*F*_c_^2^)/3
1421 reflections	(Δ/σ)_max_ = 0.001
95 parameters	Δρ_max_ = 0.39 e Å^−^^3^
0 restraints	Δρ_min_ = −0.49 e Å^−^^3^

### Fractional atomic coordinates and isotropic or equivalent isotropic displacement parameters (Å^2^)

**Table:**

	*x*	*y*	*z*	*U*_iso_*/*U*_eq_	
Br1	0.14837 (3)	0.15858 (5)	0.213462 (8)	0.02034 (10)	
O1	−0.1279 (2)	0.8670 (4)	0.04381 (7)	0.0247 (3)	
H1	−0.132 (4)	0.989 (9)	0.0131 (14)	0.042 (8)*	
N1	0.0488 (2)	0.7706 (5)	0.05189 (7)	0.0192 (3)	
C1	0.3131 (3)	0.2643 (5)	0.16293 (8)	0.0181 (4)	
C2	0.4830 (3)	0.1467 (5)	0.17939 (9)	0.0207 (4)	
H2	0.5131	0.0239	0.2144	0.025*	
C3	0.6078 (3)	0.2117 (6)	0.14383 (10)	0.0226 (4)	
H3	0.7241	0.1334	0.1545	0.027*	
C4	0.5625 (3)	0.3910 (6)	0.09268 (10)	0.0228 (4)	
H4	0.6480	0.4358	0.0684	0.027*	
C5	0.3930 (3)	0.5045 (5)	0.07699 (8)	0.0210 (4)	
H5	0.3634	0.6248	0.0417	0.025*	
C6	0.2639 (3)	0.4463 (5)	0.11190 (8)	0.0175 (4)	
C7	0.0852 (3)	0.5747 (5)	0.09540 (8)	0.0188 (4)	
H7	−0.0037	0.5116	0.1172	0.023*	

### Atomic displacement parameters (Å^2^)

**Table:**

	*U*^11^	*U*^22^	*U*^33^	*U*^12^	*U*^13^	*U*^23^
Br1	0.02289 (14)	0.02016 (15)	0.01905 (14)	0.00002 (7)	0.00669 (9)	0.00319 (7)
O1	0.0177 (7)	0.0297 (9)	0.0269 (8)	0.0036 (6)	0.0038 (6)	0.0083 (6)
N1	0.0174 (8)	0.0186 (8)	0.0213 (8)	0.0010 (7)	0.0028 (6)	−0.0004 (7)
C1	0.0213 (9)	0.0153 (9)	0.0184 (9)	−0.0018 (8)	0.0054 (7)	−0.0017 (8)
C2	0.0233 (10)	0.0184 (10)	0.0200 (10)	0.0003 (7)	0.0015 (8)	−0.0002 (7)
C3	0.0163 (9)	0.0227 (10)	0.0282 (11)	0.0013 (8)	0.0012 (8)	−0.0048 (8)
C4	0.0217 (10)	0.0243 (11)	0.0237 (10)	−0.0050 (8)	0.0081 (8)	−0.0046 (8)
C5	0.0239 (10)	0.0200 (10)	0.0193 (9)	−0.0017 (8)	0.0041 (7)	0.0001 (8)
C6	0.0194 (9)	0.0146 (9)	0.0183 (9)	−0.0023 (7)	0.0023 (7)	−0.0028 (7)
C7	0.0200 (9)	0.0183 (9)	0.0184 (9)	−0.0020 (8)	0.0038 (7)	−0.0004 (8)

### Geometric parameters (Å, °)

**Table:**

Br1—C1	1.9093 (19)	C3—C4	1.388 (3)
O1—N1	1.406 (2)	C3—H3	0.9500
O1—H1	0.86 (3)	C4—C5	1.384 (3)
N1—C7	1.277 (3)	C4—H4	0.9500
C1—C2	1.394 (3)	C5—C6	1.400 (3)
C1—C6	1.395 (3)	C5—H5	0.9500
C2—C3	1.389 (3)	C6—C7	1.471 (3)
C2—H2	0.9500	C7—H7	0.9500
			
N1—O1—H1	100 (2)	C5—C4—H4	120.0
C7—N1—O1	111.47 (16)	C3—C4—H4	120.0
C2—C1—C6	122.18 (18)	C4—C5—C6	121.63 (19)
C2—C1—Br1	116.55 (15)	C4—C5—H5	119.2
C6—C1—Br1	121.26 (15)	C6—C5—H5	119.2
C3—C2—C1	119.08 (19)	C5—C6—C1	117.05 (18)
C3—C2—H2	120.5	C5—C6—C7	121.12 (18)
C1—C2—H2	120.5	C1—C6—C7	121.83 (18)
C2—C3—C4	120.03 (19)	N1—C7—C6	120.37 (18)
C2—C3—H3	120.0	N1—C7—H7	119.8
C4—C3—H3	120.0	C6—C7—H7	119.8
C5—C4—C3	120.0 (2)		
			
C6—C1—C2—C3	−0.2 (3)	C2—C1—C6—C5	0.7 (3)
Br1—C1—C2—C3	179.03 (15)	Br1—C1—C6—C5	−178.50 (15)
C1—C2—C3—C4	−0.1 (3)	C2—C1—C6—C7	−178.85 (19)
C2—C3—C4—C5	−0.2 (3)	Br1—C1—C6—C7	2.0 (3)
C3—C4—C5—C6	0.7 (3)	O1—N1—C7—C6	−179.41 (17)
C4—C5—C6—C1	−0.9 (3)	C5—C6—C7—N1	−7.1 (3)
C4—C5—C6—C7	178.62 (19)	C1—C6—C7—N1	172.4 (2)

### Hydrogen-bond geometry (Å, °)

**Table:**

*D*—H···*A*	*D*—H	H···*A*	*D*···*A*	*D*—H···*A*
O1—H1···N1^i^	0.86 (3)	1.98 (3)	2.802 (2)	159 (3)

Symmetry codes: (i) −*x*, −*y*+2, −*z*.

## References

[bb1] Agilent (2010). *CrysAlis PRO* Agilent Technologies, Yarnton, England.

[bb2] Barbour, L. J. (2001). *J. Supramol. Chem.* **1**, 189–191.

[bb3] Jin, J., Li, Y., Wang, Z.-J., Qian, W.-X. & Bao, W.-L. (2010). *Eur. J. Org. Chem.* pp. 1235–1238.

[bb4] Schnekenburger, J. (1973). *Fresenius Z. Anal. Chem.* **263**, 23–26.

[bb5] Sheldrick, G. M. (2008). *Acta Cryst.* A**64**, 112–122. 10.1107/S0108767307043930 18156677

[bb6] Wang, X.-Z., Jia, J., Zhang, Y., Xu, W.-R., Liu, W., Shi, F.-N. & Wang, J.-W. (2007). *J. Chin. Chem. Soc. (Taipei, Taiwan)*, **54**, 643–652.

[bb7] Westrip, S. P. (2010). *J. Appl. Cryst.* **43**, 920–925.

